# Inroads into Equestrian Safety: Rider-Reported Factors Contributing to Horse-Related Accidents and Near Misses on Australian Roads

**DOI:** 10.3390/ani5030374

**Published:** 2015-07-22

**Authors:** Kirrilly Thompson, Chelsea Matthews

**Affiliations:** 1 The Appleton Institute, CQUniversity, 44 Greenhill Road, Wayville, SA 5034, Australia; 2 School of Natural and Built Environments, Civil Engineering, University of South Australia, Adelaide, SA 5000, Australia

**Keywords:** horse-rider, road safety, decision-making vehicle, risk, Australia

## Abstract

**Simple Summary:**

Riding horses on roads can be dangerous, but little is known about accidents and near misses. To explore road safety issues amongst Australian equestrians, we conducted an online survey. More than half of all riders (52%) reported having experienced at least one accident or near miss in the 12 months prior to the survey, mostly attributed to speed. Whilst our findings confirmed factors identified overseas, we also identified issues around road rules, hand signals and road rage. This paper suggests strategies for improving the safety of horses, riders and other road users.

**Abstract:**

Horse riding and horse-related interactions are inherently dangerous. When they occur on public roads, the risk profile of equestrian activities is complicated by interactions with other road users. Research has identified speed, proximity, visibility, conspicuity and mutual misunderstanding as factors contributing to accidents and near misses. However, little is known about their significance or incidence in Australia. To explore road safety issues amongst Australian equestrians, we conducted an online survey. More than half of all riders (52%) reported having experienced at least one accident or near miss in the 12 months prior to the survey. Whilst our findings confirm the factors identified overseas, we also identified issues around rider misunderstanding of road rules and driver misunderstanding of rider hand signals. Of particular concern, we also found reports of potentially dangerous rider-directed road rage. We identify several areas for potential safety intervention including (1) identifying equestrians as vulnerable road users and horses as sentient decision-making vehicles; (2) harmonising laws regarding passing horses; (3) mandating personal protective equipment; (4) improving road signage; (5) comprehensive data collection; (6) developing mutual understanding amongst road-users; (7) safer road design and alternative riding spaces; and (8) increasing investment in horse-related safety initiatives.

## 1. Introduction

The equine industry is essential to the social and economic wellbeing of Australia. “It is estimated to generate approximately AUS $9 billion each year and employ tens of thousands of people” [1]. About one third of this contribution is attributed to the non-racing sector [2], in which a quarter of a million Australians participate [3]. As well as contributing to the Australian economy, horse ownership and interactions provide numerous benefits for physical, psychological and social health [4]. However, these benefits come at tragic human cost. Approximately 20 Australians die from horse-related accidents per year [5]. “One worker is hospitalised each day in Australia due to a horse related injury. For every worker injured another nine non workers are injured” [6] (p. 2). The repercussions can be tragic: “between July 2000 to June 2012, 98 horse-related deaths occurred” (NCIS [6], p. 25). 

Horse riding is undoubtedly a “high risk interspecies sport” [7] and any interaction with horses is dangerous [8]. From the ground, horses can injure humans through biting, kicking and crushing. Even when riding in company, riders can be kicked by other horses. Riders can be transported at speeds of up to 60 km/h with their heads raised three metres above the ground. Upon falling, they may be crushed by their half tonne mount or trampled by other horses (as is common amongst jockeys). It is no surprise that recent calls have been made for a greater understanding of the objective and subjective elements of horse-related risk and equestrian risk-perception [9]. 

When riders and horses interact on public roads shared with other road users, their vulnerability to injury or death is magnified. Unlike passengers in vehicles, riders are unrestrained. They can be hit by vehicles moving at high speeds, and/or thrown into oncoming traffic, jeopardising the safety of other road users. Although the definition of vulnerable road user (VRU) specifically mentions “pedestrians, pedal cyclists and motorcyclists”, a horse/rider could easily be included due to their lack of a “hard metal shell” and their sensitivity to injury in the event of a collision or incident. VRUs are thought to be the “most sensitive to road injury” [10] (p. 1). Five interrelated factors contribute to VRU near misses and accidents: speed, proximity, visibility, conspicuity and mutual misunderstanding. 

The sudden or close passing of a vehicle may trigger a horse’s dangerous flight response, causing them to bolt blindly forward or veer into traffic [11]. The main causes of 17 horses being killed in the United Kingdom in 2011 following collisions with cars were the “vehicle travelling too close/at speed or the horse becoming spooked” [12]. Sometimes the cause of a near miss or accident arises from a lack of visibility, referring to a drivers’ “range of unobstructed vision” [13]. This may be due to a driver rounding a blind corner on the road and quickly coming upon a horse rider. A lack of conspicuity can also be a problem, referring to horse and rider being “clearly discernible” [13]. A driver may not see the horse/rider due to shadows, glare, clothing/coat colour of the horse and rider or other factors. Whilst research in the UK found no significant relationship between the wearing of fluorescent or reflective clothing and the number of near misses experienced by a rider, it did find a significant relationship between riders wearing lights and the incidence of near misses [14]. Riders wearing some form of lights experienced significantly less near-misses than other riders, whether wearing fluorescent/reflective clothing or not. A study from the UK suggests that the root cause of accidents between horse-riders and other road-users may be due to differences in hazard and risk perception, attributed to a lack of empathy. The researchers found that “drivers with horse riding experience or those with family or close friends who rode horses, showed greater ability to consider the scene from both perspectives” [15].

Whilst a UK survey of horse riders found that 60.3% of participants (n = 257) had experienced at least one near miss or accident in the year prior [14], data on the incidence of horse-related road accidents in Australia is limited. One reason for this is variable detail in admissions data from hospitals, although one study estimated that at least 8% of the 20 horse-related deaths each year in Australia occurred on roads (Cripps 2000) [5]; another is the inconsistent recording of large animal rescues by emergency services [16,17], especially those involving extricating horses from vehicle wrecks. Where police records are kept, European research has highlighted massive underreporting of VRU injury in police reports, sometimes as low as 12% [13]. However, the issue of horse-related road accidents is of increasing concern as peri-urban development in Australia could lead to a rise in the frequency of interactions between equestrians and other road users. This is particularly concerning given that data collected by the Victorian Injury Surveillance System two decades ago suggested that 16% of horse riding injuries occurred on public roads [18]. The risk is not only to equestrians. The implications for drivers can also be tragic [19].

One area of potential conflict between equestrians and other road users relates to inconsistencies in the application of national road rules, and legacy issues related to times when horses were more commonly ridden or driven on public roads and eight different sets of road rules prior to the Australian Road Rules being first published in 1999 [20]. Whilst horses are not mentioned specifically in the Australian Road rules (1999), Part 18, Division 2 comprises three “rules for people in charge of animals”. Across Australia, horses are allowed on footpaths and nature strips (subject to some conditions), may be ridden two abreast (under some conditions) and must keep left when using a roundabout while giving way to all exiting traffic. The road rules refer to horses indirectly as an animal, with the following two exceptions. The Victorian Road Rules stipulate that any rider under the age of 18 riding a horse on a road must wear a helmet while the Queensland Road Rules require that, on receiving a signal from the person in charge of a restive horse on the road, a driver must keep as far left as practicable, stop the vehicles engine and “not move the vehicle until there is no reasonable likelihood that the noise of the motor, or the movement of the vehicle, will aggravate the restiveness of the horse” [21]. 

Whilst the piecemeal research overviewed here provides some information on horse-riders as vulnerable road users, little is known about incidence and contributing factors for accidents and near misses to horse riders on Australian roads. Moreover, little is known about rider understanding of road rules. To provide a preliminary overview, horse riders were invited to take part in a pilot survey. 

## 2. Experimental Section 

### 2.1. Materials and Methods 

An online survey was conducted in consultation with the Executive Officer of the South Australian Horse Federation who commissioned the research. Surveys were designed to elicit information specifically regarding the roads that the rider frequented with their horse, any near misses or accidents in the preceding 12 months and the cause of the incidents as well as any suggestions for improving safety. The survey was open for 13 days in May 2014 and included the following six open-ended questions:

(1) Do you ride or lead horses on public roads or road related areas?
(2) Please name the roads and area in which you do the majority of your riding.
(3) Have you had any accidents or near misses on these roads in the last 12 months? If so, please describe what happened and include any suggestions for road design or rule improvement that may have helped prevent this from happening.
(4) Have you got any suggestions for updates to the Australian Road Rules you would like to see in relation to the riding or driving of horses? If so, please explain.
(5) Do you have any suggestions for road related infrastructure design or signage that would make roads more horse rider friendly?
(6) Any other suggestions you have to improve general road safety for horse riders on roads?

The survey was digitized using Survey Monkey and the link posted to the “Sa-Horse Federation” Facebook page (approximately 5000 “likes”). It was also made available on the Horse SA website with a link to the page posted in the Horse SA newsletter (approximately 1600 primary recipients).

### 2.2. Participants 

One hundred and forty seven equestrians who ride or lead horses on public roads or road related areas responded to the survey. Almost half (48%) were from South Australia, 28% were from Victoria, 12% were from New South Wales, 4% were from both Queensland and Tasmania, 2% were from Western Australia, 1% were from both the Northern Territory and England while the final 1% did not specify. Participants were not asked to provide their age or gender.

### 2.3. Analysis 

This paper reports on the responses to all questions except Question 2, which is most relevant to a local audience and the results of which could jeopardise the anonymity of respondents. Although questions 4–6 specifically asked for road rule change suggestions, signage change suggestions and any other suggestions in separate questions, respondents did not, on the whole, distinguish between these categories in their responses. These responses were therefore collated for ease of analysis and interpretation. 

Whilst respondents were restricted in the amount of open-text responses they could provide, there was sufficient data to apply a modified qualitative data analysis approach based on systematic reading of the data, recording of issues and basic organization of findings around the research questions [22]. Issues identified in open text fields were subject to descriptive statistical analyses for ease of reporting. Where informative, these statistics are illustrated and expanded upon with selected quotations reproduced verbatim from open-text response fields. 

## 3. Results

### 3.1. Rider-Reported Contributory Factors and Suggestions

Just over half of all riders (52%) reported being involved in at least one near-miss or accident within the 12 months prior to the survey. They were able to specify multiple causes for their accident(s)/near-misses. Speed was the most often cited contributor to near-misses or accidents (72%), operationalized in analysis as a vehicle passing a horse and rider at a greater speed than the rider felt safe/comfortable with. For example,

*My horse spooked at a ute [utility vehicle] that was flying towards us on a dirt road, even though I was signaling for him to slow down [an arm extended to the left and moved up and down]. He didn't stop until my horse stepped out in front of him*

*Vehicles move to other side of the road but continue to do same speed (80–100 kph) even when I’m wearing hi vis [high visability clothing] and signaling for [the] driver to slow down.*

Some riders attributed a lack of speed reduction by a driver to their lack of understanding of the unpredictably nature of horses:

*A horse can spook and put the driver is a precarious position if the horse kicks out or worse jumps on the car. Most drivers have not a clue the danger they put themselves in by passing a horse at speed*

*There seems to be a lack of understanding by the average driver about the athleticism and unpredictable nature of horses. Simply overtaking on a country road and continuing sometimes at 80+ ks is a serious risk which I experience with some regularity*

*Most drivers are not aware of how to behave around a horse and assume that if the horse is on the road that it is completely bombproof to traffic.*

Other rider-reported factors contributing to near misses were loud noises (17%), including beeping horns, revving engines, yelling; and “close” proximity (18%), defined as passing a horse and rider at a smaller distance than the rider felt safe/comfortable with. For example,

*[A] driver tried to pass me, very close, as my horse was baulking at something on the road. Fortunately she was going very slowly and although my horse backed into her car there was no major damage to either party.*

Eleven per cent of riders cited a lack of visibility, where they did not believe they were seen, due to local geography or driver inattention. Fourteen per cent cited a lack of space, where they felt forced to ride on the road due to the lack, or unsuitability, of the nature strip. Eight per cent cited noncompliance with road rules, where they believed drivers were not obeying the road rules (excluding speeding). Four per cent cited bicycles, where a bicycle approached the horse and rider from behind and passed without warning where the horse and rider were unaware of its presence, and five per cent cited the horse spooking at an animal/object not associated with the traffic. One per cent cited “other” without further specification.

As illustrated in the quotation below and throughout many of the quotations in this paper, many rider respondents reported failed attempts to signal to drivers to slow down:

*When I signal to drivers to slow down they sometimes completely ignore me even if I am clearly having trouble with my horse.*

This quotation demonstrates the ways in which some riders can assume that other road-users can interpret their horse’s behavior as not totally under control and be aware of the implications. 

Some rider respondents had been explicitly or implicitly made to feel as if they were trespassers on the road:

*... We ended up down the ditch, at which point he slammed brakes on, and abused me for being on the road on a horse, and both drivers told me horses are not allowed on the road…*

*Most drivers don't think horses and riders have a right to utilise the road and I have often been abused for being on the road.*

*Had people toot, rev engines, yell out windows causing my horse to react. Never been hit but I always try to stay well off the road edge if cars are coming.*

Seventeen per cent of rider respondents involved in near-misses or accidents reported being abused by the driver of a car. Abuse of a horse and rider was defined as the rider being yelled or cursed at, intentionally chased, having objects thrown at them or being “beeped” repeatedly.

*3X the same guy has driven at me deliberately & pulled out at the last second. 1/2 full beer bottles thrown at me & the horse, while crossing the bridge, along with having a bicycle pass me on the inside, a car sit no more than 15cms behind the horses back legs, horns tooted, abuse yelled to get off the road, all while crossing the bridge. A learner driver pass me missing my right foot by mere cms, cars speed up when over taking & or drop down a gear for more power & revved engines. So it goes on.*

One responded recalled receiving abuse from a motorcycle rider:

Have had deliberate attempts to frighten my horse (Harley Davidsons revving their motors while stationary beside me! Passengers waving and yelling as they drive past)

Another noted the difficulty of reporting offenders to police:

*If there is an incident/accident the rider is usually trying to control their horse or is on the ground after falling and there is no opportunity to get rego [registration] numbers of drivers. No point reporting the incident to the police as the driver can’t be identified. Could be worthwhile to have a study on where these incidents tend to happen though—riders might report at least locations if they knew someone/police were collating data for a study.*

### 3.2. Rider Understanding of Road Rules

Not all rider respondents knew the rules that applied to them when riding on the road. Such riders either incorrectly stated that horses have right of way or described the Queensland law where a driver must pull over and turn off their engine when a horse becomes restive. For example:

I believe a very old but valid law exists. *i.e.*, if a motorist sees a horse fractious/frightened he should pull over and turn off his engine (South Australian rider)

As noted above, this law exists only in Queensland. Even so, some variation of it was specifically mentioned by nine other non-Queenslander respondents who ride horses on roads:

*Drivers must give way and slow down and even stop if requested. The problem is our ability, or lack thereof, to enforce these rules (Victorian rider)*

*…need better publishing of the existing laws—most drivers don’t know they are required to slow down or stop if signaled (South Australian rider)*

A Tasmanian respondent was under the false impression that:

*a driver only has to do what a rider says if a horse becomes “Restive” meaning unsettled. By then it's too late!!!! The law needs to state: keep a minimum of 5 m away from the back of a horse & a minimum of 2 m away from a horse when overtaking & a speed limit of 30 km maximum when passing a horse … the law needs to be the same in all states of Australia.*

As none of the questions specifically asked respondents if they believed horses had some form of right of way on roads or if some variation of the Queensland law existed in their state, it could be surmised that at least six per cent of rider respondents not residing in Queensland or overseas demonstrated an incorrect perception about how the road rules relate to horses. 

### 3.3. Rider Suggestions for Improving Safety 

The survey returned a total of 295 suggestions for making roads safer for horses and riders. They were allocated to one of the following seven categories derived inductively from the data: education (cited by 50% of respondents), better/different signage (45%), more room for horses off roads (34%), road rule changes (27%) helmets or high visibility equipment (22%), awareness (15%) and miscellaneous other (8%).

Suggestions attributed to the category “education” related to having more information in the official drivers’ handbook, more training and “better” information more readily available. The category “awareness” refers more to raising awareness in the general population that horses are allowed to ride on roads as well as scare campaigns. These responses were clearly aimed at television, perhaps similar to the “Ride to Live” Campaign as mentioned by one respondent.

With regard to suggesting that riders should be encouraged to wear, or have to wear by law, high visibility and safety clothing of some kind, some riders believed that their experience riding on the road was more positive while they were wearing high visibility clothing.

*I believe if riders ride on the road they should wear a high vision (sic) vest. I have recently started doing this every time I leave my property gates and I feel it makes a HUGE difference to my safety.*

*Possibly consider riders wearing safety vest and/or hi viz for their horses make them more visible. I have done this a few times now and found drivers acknowledge the risk and slow down.*

**Figure 1:**
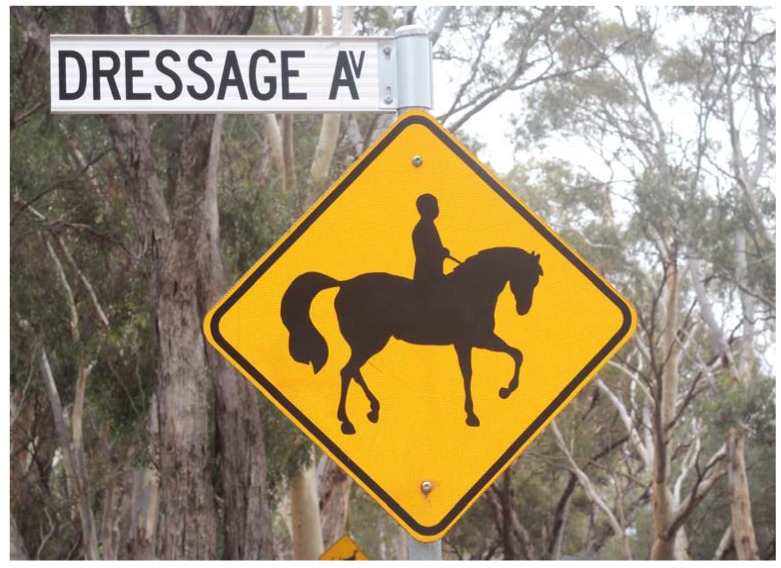
Image of sign taken at the intersection of Dressage Avenue and Pimpala Road in South Australia. Note the absence of a helmet from the visual image and a lack of any informative text. Photo Credit: Chelsea Matthews, 2015.

Regarding better/different signage, riders reinforced a perceived lack of understanding amongst drivers about the nature and behaviour of horses. That is, they considered the current signage inefficient and meaningless (see Figure 1). 

Some riders recommended the addition of specific instructions to signage:

*I think a “slow when passing” or something similar in addition to the picture would be helpful.*

*I think it could help to include “Pass Slow & Wide” with the pictured horse & rider sign commonly used. Just telling people there are horses about doesn't help them know what to do.*

*Maybe the yellow signs with horse pics [pictures] on them could include lower speed limit or state “reduce speed”.*

One respondent provided a passionate rationale for why any changes or improvements would be fortified by a public education campaign (caps in original):

*… I think the best way to achieve this is a major … road safety campaign that is put in newspapers and on the television, something particularly graphic that highlights that many of these people out there riding are young girls, mothers, daughters, brothers, sisters, uncles etc. They are PEOPLE. And they can and will die from people doing stupid things past them. There are numerous road safety campaigns for horses but they are all online and will only be seen by those people looking for them, which is mainly other horse riders. This campaign needs to reach the people who aren't looking for it, hence suggesting mass media. A recent Facebook post regarding a tabard with a built in camera has attracted numerous comments from people saying they get annoyed by horse riders and deliberately speed up to teach them a lesson and hopefully they won’t ride on the roads anymore. They need to learn that this is ILLEGAL and could not only kill the rider but kill them too. A nice image of a driver being taken to hospital and dying while a horses legs stick out his windscreen might go some way to doing the trick.*

This quotation demonstrates a riders’ awareness of the interdependence of rider, horse and driver safety. Whilst riders and drivers may seem at odds when sharing roads, they have a shared responsibility for each other’s safety.

## 4. Discussion

### 4.1. Incidents and Contributing Factors

The data suggest an incidence rate similar to the 60% reported for riders in the UK [15]. More than half (52%) of riders had experienced at least one accident or near miss within the 12 months prior to the survey. Based on this figure, and using a conservative estimate of 50% of the 226,100 Australians participating in horse-riding (ABS, 2000) [3], this would suggest that around one hundred thousand horse riders and handlers are at risk of an accident or near-miss whilst riding their horse on an Australian road.

Our findings also confirm the factors contributing to near misses and accidents identified in other countries [15], namely speed, proximity, low visibility, low conspicuity and mutual misunderstanding. Speed was cited as a contributing factor in just over half of all rider-reported near misses and accidents. In addition to these five factors, we also found evidence of rider misunderstanding of road rules and driver misunderstanding of rider hand signals. The latter is unsurprising given that hand signals used by riders to request a driver to take an action are not included in the South Australian Drivers Handbook [23] (p. 33) or the road rules of any Australian state or territory. 

Of particular concern were the experiences of road rage reported by riders. Although it may be a result of mutual misunderstanding or rider misunderstanding of road rules, it is illegal. The South Australian Drivers Handbook specifically mentions that drivers should not accelerate or rev their engine near a horse, sound their horn or make unnecessary noise and not throw objects or shout at a horse or rider [23] (p. 33). Directing road rage at horse riders can also have fatal results. By frightening horses and riders, abuse itself could contribute to an (additional) accident or near miss. For example, one incident of driver road rage directed at a horse-rider resulted in the death of a horse and the injury of a rider in Florida, America [24].

The contributing factors of speed, proximity, visibility, conspicuity and mutual misunderstanding are discussed in this paper from the perspective of VRUs. However, it should be noted that riders, other road users and horses can all contribute to, or mitigate, risks. For example, when riders ride at speed, they restrict a driver’s ability to safely reduce speed when passing. Similarly, when there is “mutual misunderstanding” between rider and horse, they are less predictable to other road users. Following research considering risk and safety as emergent properties of socio-technical networks [25], further research should consider the ways in which risk factors are distributed across the horse-rider-road-driver-car network. It is also important to recognize the perspective of each actor in the network. For horses, the visibility and conspicuity of other road users is particularly aural. This raises important concerns for electric vehicles and bicycles that are less audible and may “spook” horses more easily. 

### 4.2. Research Limitations

Findings in this paper are biased towards self-selection and the experiences of South Australian riders. Almost half (48%) of respondents resided/rode in South Australia while data from the Australian Bureau of Statistics 2000 suggests that South Australia had one of the lowest participation rates in horse activities of all states and territories in Australia at 1.4%. The Australian Capital Territory had the highest participation rate of 2.4% followed by Queensland and New South Wales at 2%. Victoria and Western Australia had participation rates of 1.8% followed by Tasmania at 1.5% while the Northern Territory was equal with South Australia at 1.4% [3]. Despite the Horse Federation of South Australia being active nationally, it is unsurprising that the majority of participants were located in South Australia. This is consistent with other national surveys promoted primarily by the Horse Federation of South Australia [26,27]. To validate the findings in this exploratory research, and determine if there are any statewide differences, further research should recruit participants from across Australia. 

Further research will need to distinguish between a near miss and an actual accident, and collect demographic data including age, gender, riding experience. As these are all important factors [28], this data should be collected in future research with a more representative sample size.

### 4.3. Potential Safety Interventions

Based on the findings from the survey and following suggestions made by riders themselves, we have identified several areas for potential safety intervention

#### 4.3.1. Identifying Equestrians on Roads as VRUs and their Horses as Sentient Decision-Making Vehicles

Terminology matters. Although riders are “vulnerable road users”, the term is not defined in Australian law, nor are VRUs referred to in any legislation with respect to road or traffic laws. The legal definition of horse riders, horse drivers (*i.e.*, cars, carriages and racing harnesses) or horse handlers at a national level as vulnerable road users could change driver perceptions, promote research and provide a rationale for funding. 

Terminology also has implications for animals, including the ways in which they are valued and prioritized relative to other lives and things [29]. This has been noted by White in relation to natural disaster response where he refers to a “strict legal categorisation of companion animals as personal property, or things, rather than legal persons” [30] (p. 381). Emergency services personnel risk their lives to save human life, property and the environment—in that order. Animals such as horses may be considered “property” or “environment”. The choice affects where attention is placed in preventing horse-related road accidents and prioritizing resources when attending callouts or making triage decisions for people and animals. From a perspective of psychological first aid, considering the horse “just a vehicle” may trivialize the trauma suffered by those at the scene and even compound the distress reported by some responders attending accidents involving horses [17]. “Vehicalising” horses also ignores the extent to which horses are not just “things” to their humans. Consistent with Belk’s use of the term “extended self” [31,32], horses are extensions of their riders’ selves. As such, the behaviour and controllability of each individual horse is at least as diverse as that of every individual rider, driver and handler. From an actor-network theory perspective, horse-riders are sentient assemblages generated by the interactions of human, technology and animal being, who often share roads with other assemblages, such as driver-cars [33]. 

Terminology is particularly important in incident analysis and accident reporting. In determining causal factors, investigators may consider road, vehicle or human factors. However, the interaction of human and vehicle factors is more straightforward when the vehicle is a car or motorbike, and much less so when the vehicle is a horse (or a donkey, mule, bullock, yak or camel). This has been demonstrated in research on horse-rider relations where subjective experiences of trust have been found to have objective consequences for riders’ risk-taking behaviours [7] and the degrees of “control” sought over their horses [34]. 

At worst, horses can be viewed as decision-making vehicles. They are like vehicles in that they transport humans and goods and are subject to road rules, but they are unlike vehicles in that they are sentient creatures capable of making their own decisions and subject to their own instincts and training. Horses (at least, well trained ones) have even been proposed as a useful metaphor for designing automated vehicles [35]. Defining horses in road rules as something more specific and sentient than just a vehicle may strengthen efforts to educate other road users about the “risky” nature and behaviour of horses—especially in relation to how horses respond to stressors. The essential differences between horse-riders, cyclists and motor vehicles could be emphasized in the development of specific road rule terminology for horses other than “vehicle” or “animal”. As there are multiple alternatives (if not just “horse”), each with different implications for the perception of horse-riders by other road-users, discursive and empirical research is required to identify and evaluate the terminology most likely to reduce horse-related near misses and accidents on public roads. 

#### 4.3.2. Harmonising Laws Regarding Passing Horses

The implementation of a law that stipulates the manner in which a driver must pass a horse being ridden, driven or led on a road may make roads safer for horse riders, drivers and handlers. One possible remedy would be to implement the Queensland law that drivers must pull over and turn off their engine if a horse becomes restive. Another option could be a law requiring drivers to pull over and turn off their engine. Although the respondent quoted above was incorrect about the existence of such a law in Tasmania, they raise a valid point; By the time a horse becomes “restive”, both the horse and rider have already been placed in danger. Her suggestions for keeping a 5 m buffer behind a horse and 2 m to the side with a speed limit of 30 km/h for overtaking could prove quite effective for reducing the instances of near misses due to horses being frightened or spooked by fast passing or close vehicles but may prove impractical in some cases. However, reducing speed may not always be a possible or safe option, such as when a vehicle on a blind corner sees a horse after entering a turn at speed, or on the rare occasion that there are horses on both sides of a road.

One practical solution with respect to law changes could be to require that drivers slow down when passing a horse and give the horse a lateral buffer where possible when passing. Further research is needed to determine a buffer distance and safe and practical speed. They may differ for large vehicles and trucks that can scare horses if they pass too slowly and/or if air brakes are applied. Harmonising road rules across Australia’s states and territories may also reduce general misunderstanding or confusion about the legal specifications for the interactions of horses and other road users. Riders could be kept informed of road rules through a test similar to the British Horse Society’s “Riding and Road Safety Test”.

#### 4.3.3. Mandating Personal Protective Equipment

Horse riding helmets are one form of “shell” that horse riders drivers and handlers can wear as a proven form of protection whilst riding, driving or leading horses. A 1995 draft of the Proposed Australian Road Rules included “requirements that all horse riders wear a helmet, reflectors when riding at night and be allowed to use footpaths and nature strips” [36]. However, with the exception of riders 18 years and under on Victorian roads, wearing helmets whilst riding on private property or public roads is still voluntary in Australia. Whilst wearing appropriate footwear when riding is a widespread practice, the low use of helmets when riding is alarming. For instance, a study of fall related injuries in Australian agriculture reported that 79% of hospital presentations “did not report use of a safety device at the time of injury” and “only 18% of injury presentations resulting from a height-related fall reported wearing head protection” [37]. Twenty years ago, researchers suspected that “[t]he promotion of equestrian helmets in Australia is likely to need a similar approach to that used to promote and effectively introduce mandatory helmet wearing” [36]. Given the multiple psychological, social and cultural barriers to increasing the voluntary use of helmets amongst horse riders [38], compulsory use and appropriate enforcement of wearing helmets on public roads should be seriously considered in Australia. 

Despite research finding no relationship between the use of high visibility clothing and reduced risk [14], some respondents in this Australian survey recalled more positive road riding experiences while wearing high visibility clothing. Their perception requires further investigation; especially as those who wear high visibility clothing may be more predisposed to “safer” riding practices in general. The use of “GoPro” style camcorders should also be considered as a form of personal protective equipment. Research is required to determine their impact on discouraging drivers from dangerous behavior such as road-rage, as well as their usefulness in recording number plates of dangerous drivers.

#### 4.3.4. Improving Road Signage 

Forty seven per cent of respondents recommended more or improved road signage. Road signage in Australia with respect to horse riders is fairly limited and usually consists simply of a yellow sign with a black horse and rider silhouette (Figure 1, above). Reviewing the current signage for horses on roads and making it more informative for drivers could be a very positive step forward for horse and rider safety. By providing some indication of how the horse needs to be treated when approaching and passing, drivers should be better able to react appropriately when they see a horse. One very simple addition to current horse signage could be the phrase “pass wide and slow”, as is currently available on some specialty high visibility rider clothing. Similarly, a diagram showing the dimensions of a buffer zone around the horse for passing drivers could inform drivers about how to pass horses more safely. 

The location of signage also needs to be reviewed as many respondents reported that there were not enough signs in their area or that they had asked for some/more signage and it had not been provided. Councils could be encouraged to provide signage for riders, particularly in areas with a higher concentration of horse traffic. 

#### 4.3.5. Data Collection

There is no comprehensive data on horse-related injury in Australia, let alone that occurring on roads. More rigorous data collection is required to:

(a) Determine the distribution, frequency and consequence of horse-related near-misses and accidents
(b) Identify at-risk rider and driver groups, or high risk locations
(c) Justify and prioritise interventions
(d) Evaluate interventions

The implementation of a mapping system over a number of years could be of assistance, similar to that used by the British Horse Society. In 2010, they launched a website dedicated solely to equestrian safety where riders can report incidents including the location and the type of incident as well as find further advice and information on road safety. Data obtained from this reporting system is intended to “*lobby those in power to make the changes that are required to ensure riding is safer for all*” [39].

#### 4.3.6. Developing Mutual Understanding amongst Road-Users

As noted above, drivers often misperceive the amount of control that riders have over their horses [15], and riders assume that other road users can interpret horse behaviour. The present study reinforces the existence of a general mutual misunderstanding between riders and drivers that in some instances leads to road rage. We also identified a lack of rider understanding of road rules. Although clarified or additional laws and signage changes may assist with reducing horse-related road incidents, these legal and technical interventions would be more successful in combination with social, educational and behaviour change interventions. Formalising and effectively communicating protocols for safely interacting with horses on roads will require engagement with all relevant stakeholders and end-users. Focus group research similar to that conducted in the UK [15], may enable the identification of important barriers and enablers to mutual understanding and the adoption of safer behaviours by all road users, for the benefit of all road users. Findings could be used to design fact sheets, infographics or other information-based safety interventions.

Interventions could draw from and enhance social connectedness rather than reinforce division and competition amongst road users. Thompson proposed the “pet as protective factor” principle to engage pet owners in activities that can increase their chances of surviving a natural disaster [40]. The principle aims to reconfigure pet ownership from a risk factor for survival to a protective factor by leveraging from people’s desire to save their pets. As the preparations required to improve an animal’s chance of surviving a natural disaster are almost always the same ones that improve human survival, this provides an opportunity for engaging with those members of the population who wouldn’t make natural disaster preparations for themselves. And for those pet guardians who wouldn’t deliberately seek information on natural disaster preparedness, animal-related social networks may provide a conduit for the dissemination of that information [41]. More recently, the pet as protective factor has been refined through “who depends on you?” messaging and extended from a premise of “save your pets and you might save yourself too” to one of “save yourself so you can take care of your pets now and after a disaster or emergency” [42]. 

Research on the risk perception of riders in high-risk equestrian sports found that riders were more likely to worry about their horses being injured than themselves [7]. This suggests that horse riders, handlers, drivers and guardians could be motivated to engage in personal injury prevention measures on public roads for the benefit of their horses. Due to the taken for granted riding relationship in human-horse relationships [8], the “pets as protective factor” principle and “who depends on you?” message [42] take on multiple meanings. First, riders could be more likely to engage in precautionary behaviours if the benefits to their horses are emphasized. Second, precautionary and “personal protective behaviour” could include improved horse training and education to increase rider control and/or reduce horse unpredictability [43]. In this regard, a well trained and understood horse could in certain circumstances be a literal form of rider protections. Third, horses depend on riders to keep them safe on the roads, and they depend on riders keeping themselves safe so that they can continue to care for their horses. Finally, road users depend on one another. Emphasizing the ways in which horses and their riders depend on drivers of cars, and vice versa, could create a platform of care rather than conflict.

#### 4.3.7. Safer Road Design and Alternative Riding Spaces

Increasing population growth, urban densification and peri-urban development could increase interactions between multiple road users. In addition to improving the safety of those interactions on public roads, there is a need to consider the provision of alternative spaces for horses. These could range from specially marked laneways or “bridleways” for horses in high horse-traffic areas to open spaces including ovals, trails, stock routes, parks and forests. Where open spaces are shared with other users such as cyclists, motorised trail bikes, walkers and people with dogs, there is still a need to promote mutual understanding, respect and rules of engagement. In a previous survey of horse owners in South Australia, rider access to national parks was raised as a safety control to keep riders away from roads [44]. However, sharing parks with off-road motorbike users was also noted as risky for horse riders, suggesting the same need for protocols around multiple user interactions on trails and in parks as identified for public roadways. Furthermore, alternative riding spaces require horse-friendly design, regular maintenance and possible biosecurity controls where horse hooves and manure may introduce germs or weeds (as do walking shoes, bicycle tyres and dog faeces). The issue of horse access to parks and other public spaces should also be seen as a safety issue.

#### 4.3.8. Increasing Investment in Horse-Related Safety Initiatives

All of the aforementioned measures require resources. Despite an average of 20 deaths per year in Australia’s equestrian sector being a figure that would be completely unacceptable in any other industry or activity, there is currently little financial support of horse-related safety research or interventions. By contrast, an average of 1.7 people die each year in Australia from shark-related injury [45], yet the State Government of Western Australia invested AUS $22 million in its Shark Hazard Mitigation Program [46]. To secure much-needed funding, the Australian equestrian industry must draw attention to the scale, severity and consequence of horse-related accident and injury. Whilst effective technical interventions such as helmets, back protectors and high visibility clothing are freely available and affordable, they lack equally effective legal and behaviour change interventions to mandate or increase their adoption. One avenue for acquiring funds to safeguard the people sustaining Australia’s $9 billion equestrian industry [1] could be the introduction of a national horse registration system. This could also help to address the lack of important data noted above.

## 5. Conclusions 

Insufficient action is being taken to improve the safety of around a quarter of a million horse people in Australia in general, let alone their safety whilst sharing public roads. This places other road users and pedestrians at risk of serious injury or worse. By reporting findings from an exploratory study of 147 equestrians who ride or lead their horses on public roads or road-related areas, we were able to identify that just over half of those riders had experienced a near miss or accident in the 12 months preceding the survey, and that speed was identified as a contributing factor in almost three quarters of those cases. As road incidents involving horses can put the lives of all road users at risk, there is an urgent need for more research to mitigate horse-related road accidents and near misses. This paper identified several areas for potential risk management spanning technical, social, legal, informational, behavioural and environmental interventions. The safety of Australia’s horse people and those around them would be well supported by the establishment of a national task force on equestrian safety with a working group on road safety. To address the complexity of the issue collaborations should be established between safety scientists, horse behaviourists, behaviour change experts, social marketers, civil engineers, town planners, police and emergency services, large animal rescuers and relevant motor accident authorities, not to mention horse riders, drivers and handlers themselves. 
